# Population genomic insights into the domestication of *Brassica juncea* var. *tumida*

**DOI:** 10.1093/hr/uhaf298

**Published:** 2025-11-05

**Authors:** Hao Wang, Xu Cai, Zhongrong Guan, Jian Wu, Lisha Peng, Wenping Li, Ling Rao, Shiwei Yang, Zhaorong Zhang, Xingxing Zhang, Yonghong Fan, Xiaowu Wang, Jinjuan Shen

**Affiliations:** Zhacai (Tuber Mustard) Research Institute, Southeast Chongqing Academy of Agricultural Sciences (Fuling Academy of Southwest University), Chongqing 408000, China; Sichuan-Chongqing Joint Key Laboratory of Science and Technology Innovation for Chinese Pickled Vegetables, Chongqing, China, Southeast Chongqing Academy of Agricultural Sciences, Chongqing 408000, China; State Key Laboratory of Vegetable Biobreeding, Institute of Vegetables and Flowers, Chinese Academy of Agricultural Sciences, Beijing 100081, China; Zhacai (Tuber Mustard) Research Institute, Southeast Chongqing Academy of Agricultural Sciences (Fuling Academy of Southwest University), Chongqing 408000, China; Sichuan-Chongqing Joint Key Laboratory of Science and Technology Innovation for Chinese Pickled Vegetables, Chongqing, China, Southeast Chongqing Academy of Agricultural Sciences, Chongqing 408000, China; State Key Laboratory of Vegetable Biobreeding, Institute of Vegetables and Flowers, Chinese Academy of Agricultural Sciences, Beijing 100081, China; Zhacai (Tuber Mustard) Research Institute, Southeast Chongqing Academy of Agricultural Sciences (Fuling Academy of Southwest University), Chongqing 408000, China; Sichuan-Chongqing Joint Key Laboratory of Science and Technology Innovation for Chinese Pickled Vegetables, Chongqing, China, Southeast Chongqing Academy of Agricultural Sciences, Chongqing 408000, China; Zhacai (Tuber Mustard) Research Institute, Southeast Chongqing Academy of Agricultural Sciences (Fuling Academy of Southwest University), Chongqing 408000, China; Sichuan-Chongqing Joint Key Laboratory of Science and Technology Innovation for Chinese Pickled Vegetables, Chongqing, China, Southeast Chongqing Academy of Agricultural Sciences, Chongqing 408000, China; Zhacai (Tuber Mustard) Research Institute, Southeast Chongqing Academy of Agricultural Sciences (Fuling Academy of Southwest University), Chongqing 408000, China; Sichuan-Chongqing Joint Key Laboratory of Science and Technology Innovation for Chinese Pickled Vegetables, Chongqing, China, Southeast Chongqing Academy of Agricultural Sciences, Chongqing 408000, China; Zhacai (Tuber Mustard) Research Institute, Southeast Chongqing Academy of Agricultural Sciences (Fuling Academy of Southwest University), Chongqing 408000, China; Sichuan-Chongqing Joint Key Laboratory of Science and Technology Innovation for Chinese Pickled Vegetables, Chongqing, China, Southeast Chongqing Academy of Agricultural Sciences, Chongqing 408000, China; Zhacai (Tuber Mustard) Research Institute, Southeast Chongqing Academy of Agricultural Sciences (Fuling Academy of Southwest University), Chongqing 408000, China; Sichuan-Chongqing Joint Key Laboratory of Science and Technology Innovation for Chinese Pickled Vegetables, Chongqing, China, Southeast Chongqing Academy of Agricultural Sciences, Chongqing 408000, China; Zhacai (Tuber Mustard) Research Institute, Southeast Chongqing Academy of Agricultural Sciences (Fuling Academy of Southwest University), Chongqing 408000, China; Sichuan-Chongqing Joint Key Laboratory of Science and Technology Innovation for Chinese Pickled Vegetables, Chongqing, China, Southeast Chongqing Academy of Agricultural Sciences, Chongqing 408000, China; Sichuan-Chongqing Joint Key Laboratory of Science and Technology Innovation for Chinese Pickled Vegetables, Chongqing, China, Southeast Chongqing Academy of Agricultural Sciences, Chongqing 408000, China; State Key Laboratory of Vegetable Biobreeding, Institute of Vegetables and Flowers, Chinese Academy of Agricultural Sciences, Beijing 100081, China; Zhacai (Tuber Mustard) Research Institute, Southeast Chongqing Academy of Agricultural Sciences (Fuling Academy of Southwest University), Chongqing 408000, China; Sichuan-Chongqing Joint Key Laboratory of Science and Technology Innovation for Chinese Pickled Vegetables, Chongqing, China, Southeast Chongqing Academy of Agricultural Sciences, Chongqing 408000, China

## Abstract

*Brassica juncea* var. *tumida*, commonly known as Zha Cai, is a pickled stem mustard widely cultivated in southern China. Its most distinctive trait is the swollen stem, which serves as the primary economic organ for harvest. However, the origin and domestication history of *tumida* remain unclear, hindering genetic improvement and molecular breeding efforts. Here, we assembled a chromosome-level genome of the landrace ‘YAXY’ from Chongqing—the center of *tumida* diversity—totaling 909.1 Mb with a contig N50 of 4.17 Mb. We also collected and resequenced 203 *tumida* accessions across southern China. By integrating the ‘YAXY’ reference genome with population data, we generated the first comprehensive *tumida* variation dataset, comprising 1.38 million single-nucleotide polymorphisms (SNPs) and 0.27 million insertions and deletions (InDels). Joint analysis of the newly sequenced *tumida* population and 504 public *B. juncea* datasets revealed that *tumida* and leafy types from southern China share a common origin from local oilseed mustard. *Tumida* domestication was accompanied by a strong genetic bottleneck. Additionally, we conducted genome-wide association studies (GWAS) for 21 agronomic traits and identified candidate genes linked to key domestication traits in *tumida*. For the swollen stem trait, selective sweep and GWAS analyses jointly identified candidate genes likely involved in lignification. Transcriptome data showed consistent differential expression of *BjuA05g15010*, the *Arabidopsis SAGL1* ortholog, across all swelling stages, suggesting a key role in stem morphogenesis. Collectively, our findings shed light on *tumida* evolution and provide valuable genomic resources and candidate genes to support genetic research and breeding in *B. juncea*.

## Introduction

*Brassica juncea* (L.) Czern. & Coss. is an important cruciferous crop cultivated worldwide, holding significant agricultural and economic value [1]. During long-term domestication and breeding, it has gradually differentiated into four major cultivated types: oilseed mustard, root mustard, leaf mustard, and stem mustard [2, 3]. Among these, tumorous stem mustard (*Brassica juncea* var. *tumida* Tsen et Lee, hereafter referred to as *tumida*) represents a distinctive Chinese variety of stem mustard. Its characteristic tumorous swollen stem serves as the primary economic organ [4–6]. Although this swollen stem is a key domestication trait directly governing both yield and quality, the domestication history of *tumida* remains poorly understood, severely constraining genetic improvement and molecular breeding advancements.

Excessive lignification in the swollen stem presents a major challenge in *tumida* breeding. This process is predominantly regulated by secondary cell wall (SCW) biosynthetic genes [7]. Notably, germplasm with stronger lignification is frequently observed to exhibit a thicker Stem Peripheral Tissues (SPT). Although lignification enhances mechanical strength and abiotic stress tolerance in plants, its excessive accumulation in the edible swollen stem of *tumida* adversely affects horticultural quality, particularly in terms of texture, crispness, and palatability [8]. Consequently, breeders have empirically employed fresh weight of the SPT as a high-throughput phenotypic proxy to select germplasms with potentially reduced lignification. However, this empirical strategy suffers from substantial limitations in both accuracy and efficiency. Critically, the potential roles of SCW biosynthetic genes in regulating lignification during swollen stem development in *tumida* remain largely uncharacterized, which severely impedes targeted genetic improvement.

Recent genomic studies have begun to reveal genomic features of *tumida*. Among these, a chromosome-level genome assembly for a *tumida* (‘T84-66’, originating from Zhejiang, China) revealed structural variations, such as a 13 Mb deletion on chromosome A06 involving *HSP20* and *TGA1* genes, contributing to divergence between oilseed and vegetable types [9]*.* GWAS and transcriptome analyses have further identified regulatory modules, such as CK2B1-E2Fa, that control stem swelling via the regulation of cell division [6]. Furthermore, population genomic analyses of 480* B. juncea* accessions revealed a West Asian origin of the species and three independent eastward domestication routes [1, 10].

Current research efforts are predominantly concentrated on dissecting the genetics of oilseed traits in *B. juncea* [9–12]. Crucially, studies specifically focused on the evolutionary history of *tumida* and the genetic mechanisms underlying lignification development within its swollen stem are notably scarce. Importantly, the reliance on a single reference genome ‘T84-66’ and lack of population genomic resources covering the diversity of *tumida* restrict systematic analysis of its allotetraploid genome [13]. This also impedes elucidation of the evolutionary trajectory of this crop. This gap fundamentally constrains progress towards molecular design breeding for *tumida* aimed at quality improvement, such as reducing lignification.

**Figure 1:**
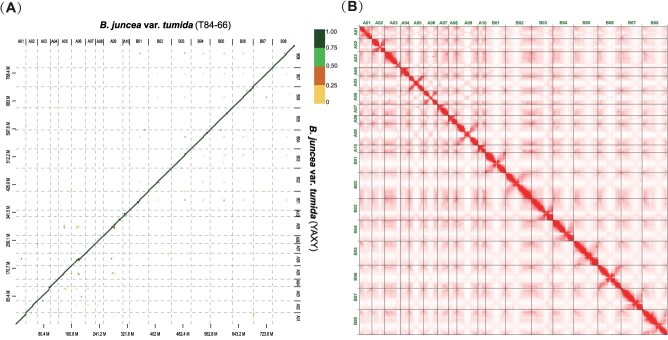
Comparison between assemblies of ‘YAXY’ and ‘T84-66’. (A) Dot-plot alignment of the ‘YAXY’ with ‘T84-66’. (B) Whole genome contacts of Hi-C data of ‘YAXY’ and ‘T84-66’ genomes.

Population genomics provides a systematic framework for deciphering the genetic architecture of crop domestication traits [14–16]. To elucidate the evolutionary history and diversity of *tumida*, this study generated a chromosome-scale *de novo* genome assembly for the landrace ‘YAXY’ (originating from Chongqing, China). We also collected and resequenced 203 *tumida* accessions from southwest China, identifying 1.38 million SNPs and 0.27 million InDels. Integrated population genetic analysis with public genomic data from 504 *B. juncea* accessions revealed that *tumida* likely originated from oilseed mustard in the southern China, underwent a genetic bottleneck during eastward dispersal, and diversified in southern China following divergence from leafy mustard ancestors. GWAS, combined with selective sweep analysis, identified candidate genes associated with key domestication traits, such as stem fresh weight and plant height. The complete list of associated loci and candidate genes is available at Figshare (https://figshare.com/s/7e8cc1eb750c4c20add7). This work systematically reconstructs the domestication history of *tumida* and provides foundational genomic resources for future genetic improvement.

## Results

### Genome assembly and annotation of *B. juncea* var. *tumida* ‘YAXY’

To elucidate the genetic basis of key domestication traits in *tumida*, we produced a chromosome-scale genome assembly for ‘YAXY’, a representative ecotype from Chongqing that has been cultivated locally for more than 30 years. Compared with the Zhejiang ecotype T84-66, YAXY exhibits earlier bolting and flowering, a larger swollen stem, and distinct differences in morphology and cultivation practices. The assembly integrated data from multiple platforms: Illumina NovaSeq 6000 short-read sequencing (137.01× coverage), PacBio Sequel II long-read sequencing (113.89×), Bionano Saphyr optical mapping (158.77×), and Hi-C chromosome conformation capture (106.55×) (Table S1). The final assembly spanned 909.10 Mb, exhibited a heterozygosity rate of 0.39%, and achieved a scaffold N50 of 57.77 Mb (Tables S2 and S3). Supported by a BUSCO completeness score of 99.7% and clear Hi-C maps (Fig. 1; Table S4), the YAXY assembly shows high quality, with additional information provided on putative centromeric and telomeric regions and assembly gaps (Tables S5–S7). In addition, we aligned the YAXY and T84-66 genomes with MUMmer4 and used SyRI to identify numerous sequence and structural variants (Table S8).

Gene prediction yielded 87 489 protein-coding genes, utilizing an evidence-based approach that integrated *ab initio* prediction, protein homology, and transcriptomic evidence. Functional annotations were assigned to 85.69% of the predicted genes using the KEGG, GO, and InterPro databases (Table S9). The reliability of the gene models was further corroborated by tissue-specific expression profiles and a high BUSCO completeness score of 98.4% for the predicted gene set (Fig. S1 and Table S4). Repetitive sequences constituted 44.09% (400.70 Mb) of the assembled genome, with transposable elements dominated by LTR retrotransposons (Table S10).

### *B. juncea* var. *tumida* genetic map with global variation resource

**Figure 2:**
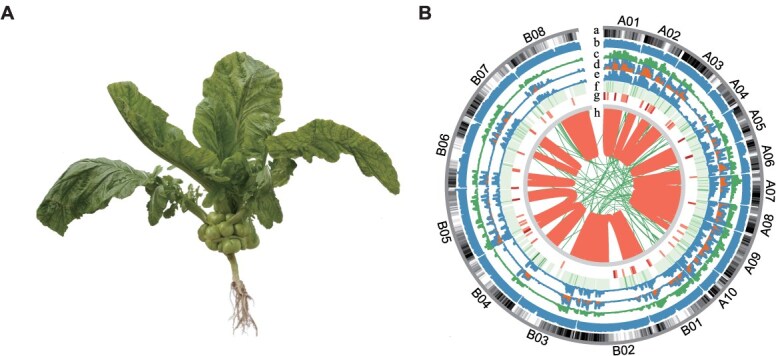
Plant morphology and genetic variation map of *B. juncea* var. *tumida.* (A) Mature plant of representative landrace ‘YAXY’. (B) Population-level genetic variation map of *tumida*. a, Gene distribution across chromosomes. b, Resequencing depth distribution. c, SNP density. d, Deletion density. e, Insertion density. f, Inversion density. g, Heatmap of Copy Number Variation (CNV) density. h, Distribution of inter-chromosomal translocations (CTX) and intra-chromosomal translocations (ITX). The genetic variation map was drawn using R ggplot2.

In this study, we collected and resequenced 203 *tumida* accessions from across southern China (Table S11), achieving an average sequencing coverage of 50.66× (Table S12). These data enabled construction of the most comprehensive *tumida*-specific genetic variation map to date (Fig. 2), comprising 1.38 million SNPs and 270 132 InDels (Table S13). Functional annotation revealed that 6.51% of coding-region SNPs (representing 386 405 missense mutations) and 1.65% of coding-region InDels (comprising 36 187 frameshift variants) showed potential functional significance (Table S14). Additionally, we integrated 504 *B. juncea* resequencing datasets from public repositories (NCBI SRA: PRJNA615316, PRJNA1148674) to construct a global variation resource encompassing 717 accessions. Through stringent four-dimensional filtering, we identified 3 561 818 high-quality SNPs and 699 689 InDels across the *B. juncea* population (Table S13).

### Population structure analysis

**Figure 3:**
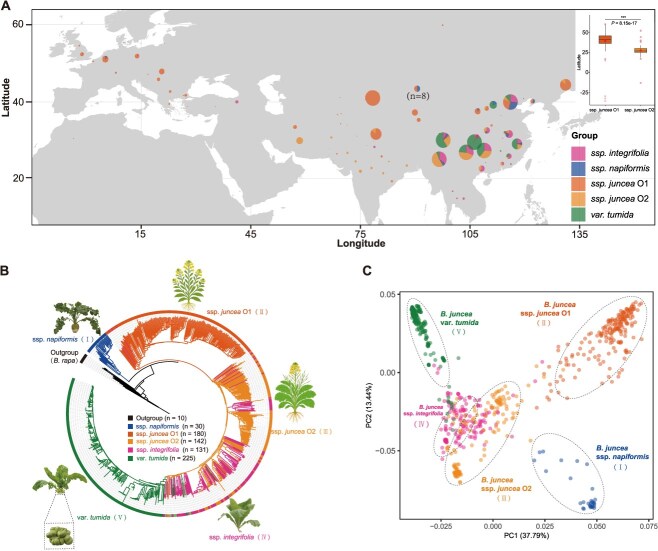
Geographic Distribution and population structure of *B. juncea* accessions. (A) Geographic distribution of *B. juncea* accessions. The geographic map was drawn using R ggplot2. Boxplot showing latitude distribution of O1 (*n* = 180) and O2 (*n* = 142) subgroups. Independent Student's *t*-test revealed significant difference (*P* < 0.001). (B) Neighbor-joining tree of *B. juncea* accessions, rooted with *B. rapa* (10 accessions) as an outgroup. Different colors indicate the accessions within different *B. juncea* type populations: clade I (root mustard, *napiformis*), clade II (Oilseed mustard O1, *juncea* O1), clade III (Oilseed mustard O2, *juncea* O2), clade IV (Leaf mustard, *integrifolia*), clade V (Stem mutard). Representative morphological pictures are displayed next to the corresponding sub-populations. (C) PCA plots showing five divergent clades of *B. juncea* accessions.

To elucidate the domestication and population structure of *tumida*, we constructed a phylogenetic tree using 3.56 million genome-wide SNPs derived from 717 globally distributed mustards accessions, totaling 18.14 Tb of raw data, with an additional 10 *B. rapa* accessions (the progenitor species of *B. juncea*) included as an outgroup (Fig. 3A and B). The analysis panel comprised 30 root mustard (*B. juncea* ssp. *napiformis*), 131 leaf mustard (*B. juncea* ssp. *integrifolia*), 322 oilseed mustard (*B. juncea* ssp. *juncea*), and 234 stem mustard (225 *B. juncea* var. *tumida,* 4 *B. juncea* ssp. *crassicaulis* and 5 *B. juncea* ssp. *gemmifera*) accessions. Rooted with *B. rapa*, the phylogeny delineated the accessions into five evolutionary clades that largely correspond to distinct morphological types.

Clade I contains *napiformis* accessions from northern and northeastern China, which constitute the earliest known domesticated group of *B. juncea* [1, 10]. Clade II comprised oilseed mustard accessions from northwestern and northern China, as well as European countries (designated O1). Clade III formed an admixed group containing oilseed mustard (from southern China and south Asia, designated O2) and *integrifolia* accessions (primarily from China). These two oilseed mustard (O1 and O2) exhibited significantly distinct latitudinal distributions (Student's *t*-test on mean latitudes, *P* = 8.15 × 10^−17^; Fig. 3A). Clade IV primarily contained *integrifolia* accessions from China and Southeast Asia. *Tumida* accessions formed a monophyletic Clade V at the terminus of the phylogeny. The short internal branch lengths within Clade V support a recent divergence event, consistent with historical records indicating that cultivation of *tumida* only began in the 18th century [1, 17].

Principal component analysis (PCA) supported the phylogenetic structure, with the first two PCA (PC1 and PC2) cumulatively explaining 51.23% of the total genetic variation (Fig. 3C). *Tumida* accessions formed a tightly clustered group distinct from other morphotypes, indicating genetic isolation and reduced diversity. In contrast, *integrifolia* accessions—positioned proximal to *tumida* in the PCA plot—exhibited partial spatial overlap with *juncea* O2 accessions, suggesting potential gene flow or historical hybridization between these groups.

**Figure 4:**
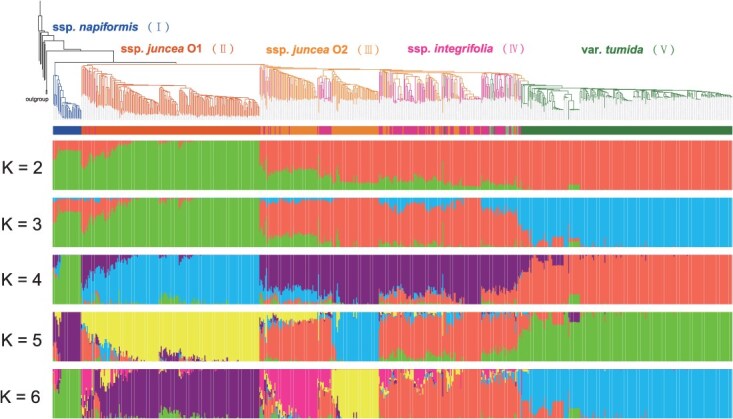
Population structure of *B. juncea* accessions. The neighbor-joining phylogeny of 717 *B. juncea* accessions and model-based clustering with K from 2 to 6. The *B. rapa* species used to root the phylogenetic tree are shown as a single branch. Branch colors indicate different groups based on the population structure.

**Figure 5:**
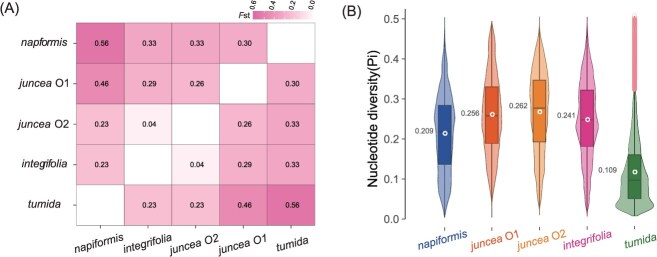
Genetic diversity in *B. juncea*. (A) Pairwise *F*st heatmap between Subgroups, Color intensity is proportional to the level of genetic differentiation, with darker red indicating higher pairwise Fst values. (B) Nucleotide Diversity across Subgroups.

Subsequently, population structure analysis at the optimal cluster number (K = 6) further resolved genetic variation in oilseed mustard (Fig. 4). It unambiguously differentiated the five principal morphological types and revealed substructure within the O2 group, which largely corresponded to two ecotypes: southern China and South Asian. *Integrifolia* accessions shared analogous ancestral components with oilseed mustard O2. Despite marked divergence in agronomic traits, genetic differentiation between *integrifolia* and O2 was negligible (*F*st = 0.04; Fig. 5A), explaining their congruent placement in phylogenetic and PCA analyses. In contrast, *tumida* exhibited a predominantly homogeneous ancestry, indicating a consolidated genetic background. Pairwise *F*st analysis confirmed significant differentiation between *tumida* and other groups (*F*st = 0.23–0.56), with minimal divergence observed between *tumida* and *integrifolia* (*F*st = 0.23), supporting their close genetic affinity as reflected by proximal clustering in PCA.

### Domestication of *B. juncea* var. *tumida*

**Figure 6:**
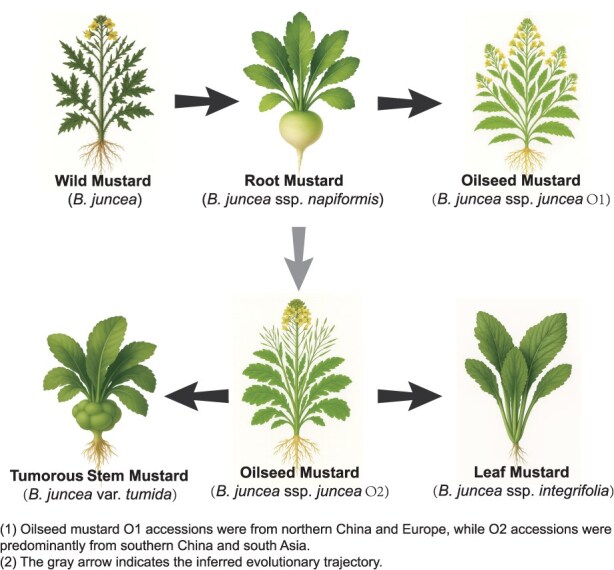
Inferred domestication route of *B. juncea* var. *tumida* based on population genetic analysis. Wild mustard underwent parallel divergence, giving rise to root mustard, oilseed mustard O1, and O2. Subsequently, oilseed mustard O2 spread eastward from the southern China, forming *integrifolia* and *tumida*.

Building on previous domestication research based on 480 accessions [1], this study greatly enriched the germplasm resources of *tumida*. We integrated evidence from population structure, genetic differentiation, and diversity analyses to propose a putative evolutionary history for *tumida* (Fig. 6): Wild mustard was domesticated in northern China and Europe, forming the oilseed mustard O1, while independently domesticated in southern China, yielding the distinct oilseed mustard O2. Subsequently, driven by domestication history and regional ecological factors, oilseed mustard O2 in south China diversified into two morphological types: leaf mustard (*integrifolia*) and tumorous stem mustard (*tumida*).

Notably, analyses of genomic variation within populations demonstrate that a severe genetic bottleneck occurred during the domestication of *tumida*. Under identical variant calling criteria (MAF > 0.05), *tumida* retained only 38.7% of species-wide genetic variation (Table S13), with SNP counts reduced to 1.38 million (38.8% of total SNPs) and InDel counts to 0.27 million (38.6% of total InDels) relative to the entire *B. juncea* population. Genome-wide scan using 200 kb sliding windows revealed extensive losses in genetic diversity in *tumida*. Median SNP density (562.33 SNPs/Mb) and InDel density (109.34 InDels/Mb) represented declines of 66.2% and 65.2%, respectively, relative to the species-wide baseline (Fig. S2). Cross-variety comparisons demonstrated that *tumida* possessed the lowest nucleotide diversity (π = 0.109), significantly lower than *napiformis* (π = 0.209), *juncea* O1 (π = 0.256), *juncea* O2 (π = 0.262), and *integrifolia* (π = 0.241) (Fig. 5B). To minimize bias from relying solely on the *tumida* reference, we reanalyzed nucleotide diversity using the *B. juncea* var. Purple-leaf Mustard (PM) reference [11], obtaining results consistent with YAXY (Fig. S3).

### Selective sweep and genome-wide association study identify key genes for swollen stem

**Figure 7:**
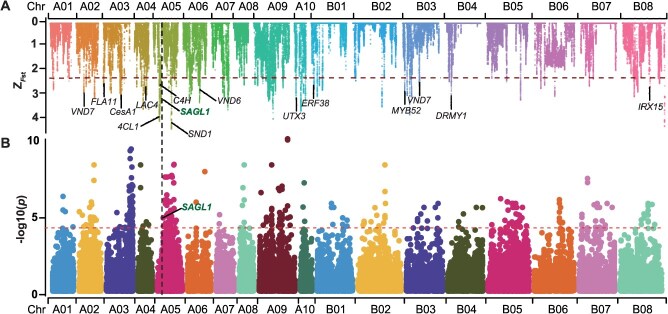
Candidate gene identification through colocalization of selective sweeps and GWAS. (A) Selective sweep analysis between *tumida* and *intergrifolia* accessions, highlighting secondary cell wall-associated genes under selection, including *SAGL1* and *CesA1*. (B) GWAS Manhattan plot for the SPT trait in *tumida*. The vertical dashed line indicates co-localization of selective sweep signals and GWAS peak at *SAGL1* locus (*BjuA05g15010*).

To elucidate selection signatures during *tumida* domestication, we identified 11 selective regions (43.02 Mb) containing 4191 genes between *tumida* and *integrifolia* through *F*st analysis (Z_*F*st_ ≥ 2.33; Table S15). Given conserved roles of SCW biosynthesis in vascular lignification [18–20], we screened 204 *Arabidopsis* SCW homologs within these regions. One-tailed Fisher’s exact test confirmed significant enrichment of 26 SCW genes (odds ratio, 2.90; 95% CI, 1.84–4.49; *P* = 1.09 × 10^−6^; Fig. 7; Tables S16 and S17).

Subsequently, genome-wide association analysis of 21 agronomic traits across 203 *tumida* accessions using 1.65 million variants identified 14 289 candidate genes (*P* ≤ 1 × 10^−4^), with maximum phenotypic variation explained (PVE) reaching 0.95 (Table S18). Notably, for the SPT trait, *BjuA05g15010*—an ortholog of the *Arabidopsis* lignin biosynthesis regulator *AtSAGL1* [21]—exhibited both strong GWAS association (*P* = 5.06 × 10^−5^) and colocalization with a selective sweep peak (Fig. 7). Convergent signatures at *BjuA05g15010* imply a potential link between lignification-associated selection and SPT variation.

### Transcriptional analysis of key SCW genes during stem swelling development

**Figure 8:**
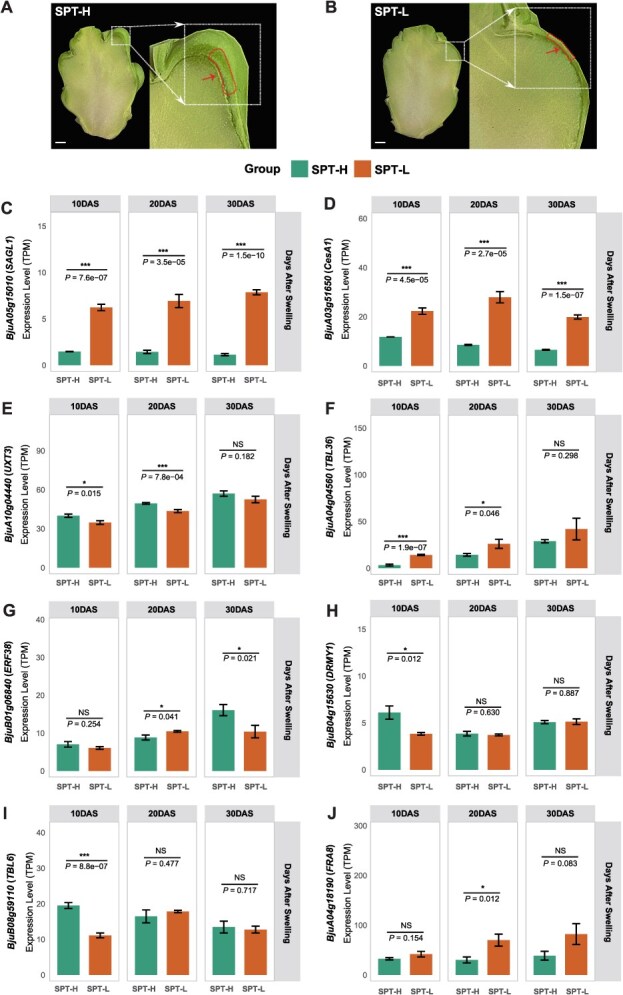
Expression levels of the SPT trait and eight secondary cell wall-associated genes during stem swelling development. Representative images of SPT-H and SPT-L phenotypes, which have high and low fresh weight of stem peripheral tissues, respectively. (A) SPT-H phenotype. (B) SPT-L phenotype. Scale bar = 1 cm. (C–J) Expression levels (TPM) of eight secondary cell wall-associated genes in SPT-H and SPT-L groups across three developmental stages (10, 20, 30 DAS): *BjuA05g15010* (C), *BjuA03g51650* (D), *BjuA10g04440* (E), *BjuA04g04560* (F), *BjuB01g06840* (G), *BjuB04g15630* (H), *BjuB08g59110* (I), and *BjuA04g18190* (J).

To investigate gene expression dynamics during stem swelling, we performed multi-stage transcriptome sequencing on *tumida* accessions representing extreme SPT phenotypes (SPT-H and SPT-L; Fig. 8A and B) at 10, 20, and 30 days after swelling (DAS). Accessions with a higher ratio of stem peripheral tissue to total swollen stem weight were defined as SPT-H, which has been empirically used in breeding as a proxy for stronger lignification, whereas those with a lower ratio were defined as SPT-L. Temporal expression profiling revealed stage-specific differential expression in 30.8% (8/26) of selective-sweep-associated secondary wall orthologs (*P* < 0.05). Among these, *BjuA05g15010* (ortholog of *Arabidopsis SAGL1*) and *BjuA03g51650* (*CesA1*) exhibited consistent differential expression across all stem swelling stages in both phenotype groups (Table S19). Population variant analysis identified two missense mutations in *BjuA05g15010*: ChrA05: 9741792 (C → A, H6Q) and ChrA05: 9743180 (A → T, I414L). Allele frequency analysis revealed that these missense alleles are common in ancestral non-swollen lineages but occur at low frequency in *tumida* (Fig. S4; Table S20). Interestingly, *sagl1* loss-of-function enhances lignification in *Arabidopsis*, whereas its overexpression suppresses this phenotype [21]. Consistently, *BjuA05g15010* exhibited significantly higher expression in SPT-L than in SPT-H (*P* < 0.01). Moreover, transcriptome data showed significantly higher expression in *tumida* than in *integrifolia* (*P* < 1 × 10^−4^; Fig. S5; Tables S21 and S23).

## Discussion

‘YAXY’ is the longest-cultivated elite landrace from Chongqing, China, representing an ecotype sharply contrasting with ‘T84-66’. We generated a chromosome-scale reference genome for ‘YAXY’ and integrated it with whole-genome resequencing data from 203 *tumida* accessions. This comprehensive approach captured the major genetic variations within *tumida*, establishing an essential foundation for deciphering its evolutionary history and facilitating genetic improvement. By integrating population genomic analyses with historical records, we propose that *tumida* and *integrifolia* likely share a common origin from oilseed mustard in southern China. Furthermore, we identified candidate genes associated with several key agronomic traits, such as fresh weight of the swollen stem, plant height, and leaf size. Our findings not only provide crucial insights into the evolution of *tumida* and the genetic basis of its important traits, but the first comprehensive *tumida* variation dataset generated herein will serve as a valuable resource for future genetic research and breeding in *B. juncea*.

### Formation and domestication of *B. juncea* var. *tumida*

*Tumida*, a variant of the ancient mustard species, has a relatively recent documented history. Its processing was first recorded in a Chinese local chronicle in 1786 as ‘a green vegetable with buds and bolts, salted and named *Wuxiang Zhacai*’ [1, 17]. This record indicates the commercial processing of *tumida* in Fuling, Chongqing at that time. To date, Fuling remains the region with the earliest historical records, the richest germplasm resources, and the largest cultivation area for this variety. Previous studies, based on morphological traits, geographical distribution patterns, and historical evidence, proposed Fuling as the center of origin for *tumida* and tentatively hypothesized its derivation from *integrifolia* [22]. Our findings provide further support and deeper insights into the domestication history of this variety. Through population genomic analyses, we reconstructed its evolutionary trajectory, revealing that both *tumida* and *integrifolia* likely share a common origin from oilseed mustard in southern China.

The most distinctive domesticated trait of *tumida* is its swollen stem. Presently, this variety is predominantly distributed in southern China. Attempts to cultivate it in other regions typically fail to develop the characteristic swollen stem, suggesting that the expression of this key trait may be under strong selection by specific climatic conditions. Furthermore, during the relatively short documented history of just over two centuries, the swollen stem, being the primary economic organ, has been subjected to persistent artificial selection for increased yield. This intensive human selection pressure is likely a major factor contributing to the significantly reduced genetic diversity observed in *tumida* compared to other mustard types. Consequently, this distinctive species has been shaped by the combined forces of its domestication history and specific ecological environment.

### Genomic insights into environmental adaptation of oilseed mustard

The two oilseed mustard subpopulations, O1 and O2, exhibit not only pronounced latitudinal differentiation, but also substantial genome-wide divergence (*F*st = 0.26; Fig. 5A), suggesting ecological adaptation to contrasting climatic zones—temperate in the north versus tropical/subtropical in the south. Notably, as the most genetically diverse and geographically widespread morphotype within *B. juncea*, oilseed mustard provides a robust system for investigating the evolutionary mechanisms of environmental adaptation. Future research should integrate genome-wide variation profiles with environmental gradient data to uncover the molecular basis of thermal adaptation. Such insights are particularly relevant to understanding plant resilience under increasingly frequent extreme climatic events [23, 24].

### Future breeding strategies in *B. juncea* var. *tumida*

The pronounced reduction in genetic diversity observed in *tumida* is likely the result of combined effects from intensive artificial selection and the constraints of localized ecological environments. This erosion of genetic variation has compressed the available genomic diversity, increased the risk of fixation of deleterious recessive alleles, and diminished the plant’s adaptive capacity to environmental fluctuations [25–27], thereby limiting the potential for further genetic improvements in agronomic performance and environmental resilience. As such, systematic conservation and efficient utilization of *tumida* germplasm resources have become imperative.

To broaden the genetic base, future breeding programs should incorporate high-diversity relatives—such as leaf mustard and oilseed mustard—into elite *tumida* backgrounds to enhance allelic richness and adaptability [28–30]. Integrating genomic selection (GS) with targeted gene editing is expected to facilitate the efficient pyramiding of favorable alleles. Although no direct correlation was detected between SPT fresh weight and lignification levels in this study, together with domestication signals detected between *tumida* and *integrifolia* and GWAS results, these findings indicate that *BjuA05g15010* may be a key gene associated with the formation of *tumida*. Multi-omics evidence suggests that *BjuA05g15010*, a secondary wall regulatory gene, likely serves as a key modulator of lignification during swollen stem development. Subsequent functional validation should involve phenotypic correlation analyses between SPT and lignification-related traits, such as lignin content and syringyl/guaiacyl (S/G) ratio, spatiotemporal characterization of SCW thickening during stem development, and targeted genome editing of *BjuA05g15010* via CRISPR/Cas9. Furthermore, the development of molecular markers linked to lignification dynamics or stem swelling phenotypes based on naturally occurring genetic variants will accelerate molecular breeding efforts aimed at improving the tumorous stem trait [31].

## Methods

### Planting and phenotyping

The reference genome assembly ‘YAXY’ and the 203 *tumida* accessions, representing a broad spectrum of phenotypic diversity across China, were collected by the Southeast Chongqing Academy of Agricultural Sciences from diverse geographic regions. Young leaf tissues were immediately flash-frozen in liquid nitrogen for subsequent DNA extraction and sequencing.

All plant materials were cultivated between September and the following January at two experimental sites: the research fields of the Southeast Chongqing Academy of Agricultural Sciences (29.90°N, 107.47°E) and the Xingguang Village Experimental Base in Longtan Town, Fuling District, Chongqing (29.44°N, 107.11°E). All experimental materials received identical field management, with 50 individual plants per accession planted at fixed row spacing. A completely randomized block design with three replicates was employed. Phenotypic evaluations were conducted synchronously at both locations. At maturity, five or more uniformly growing plants per accession were selected for trait measurement. Twenty-one morphological and yield-related agronomic traits were statistically analyzed. The majority of trait measurements strictly adhered to the standards prescribed by the Chinese Crop Germplasm Resources Information System (CGRIS; https://www.cgris.net/). For traits not covered by this system, methodological details along with the complete phenotypic dataset were deposited in the Figshare repository. To minimize error impact, outliers in phenotypic data were removed using the 3σ criterion.

### Sequencing and assembly of *B. juncea* var. *tumida* ‘YAXY’

The chromosome-level genome assembly of the ‘YAXY’ in *tumida* from the primary production region (Chongqing, China) was constructed through a multi-platform sequencing strategy. Paired-end 150 bp short reads (130.98 Gb clean data, 137.01 × coverage) from 500 bp libraries sequenced on the Illumina NovaSeq 6000 platform were used for base correction and quality assessment. Long-read sequencing with 20 kb libraries on the PacBio Sequel system (11 cells, 108.88 Gb data, 113.89× coverage) was assembled using Falcon (v2016.08; parameters: -k18 -w6 -e0.75 -M28), generating primary contigs with an N50 of 4.17 Mb. Scaffolding and chromosomal anchoring were performed using Bionano Saphyr optical maps (enzyme Nt.BspQI, 151.78 Gb data, 158.77× coverage) and Hi-C chromatin interaction data (101.86 Gb, 106.55× coverage), resulting in a final chromosome-level assembly of 909.10 Mb. Hi-C interaction patterns anchored 93.91% of sequences to 18 chromosomes, achieving a scaffold N50 of 57.77 Mb and a maximum scaffold length of 71.07 Mb. The histogram summarizes the completeness evaluation using Benchmarking Universal Single-Copy Orthologs (BUSCO v5.4.7) against the embryophyta_odb10 dataset.

### Identification of centromeres, telomeres, and assembly gaps

We aligned published *Brassica* centromeric/pericentromeric repeats (CentBr1, CentBr2, CRB, TR805, PCRBr, TR238) [32, 33] and the telomere sequence BrSTR to the YAXY assembly using LASTZ (v1.04.00). Repeat enrichment was summarized in 100-kb windows with 5-kb steps. Putative centromeric/telomeric intervals were defined by peak intensity and contiguity and finalized by manual curation. Assembly gaps were identified as contiguous tracts of Ns. Because the assembly is not T2T, these coordinates are operational approximations based on motif enrichment and are not intended to define exact physical boundaries.

### Gene prediction and functional annotation

Prior to gene prediction, whole-genome transposable element annotation and TE library construction were performed for each assembly using the EDTA pipeline (v1.8.3) [34]. Genomic sequences masked by RepeatMasker (open-4.0.7) [35] with this library served as the basis for gene predictions. The prediction strategy integrated three complementary approaches: ab initio modeling via AUGUSTUS (v3.3.3) (https://github.com/Gaius-Augustus/Augustus) and GeneMark (v4) [36]; homology-based prediction using GeneWise (v2.4.1) [37] under default parameters; and transcriptome evidence prediction derived from RNA-seq data processed by Trinity (r2013-02-25) [38] and refined with PASA (r20130425beta) [39]. Consensus gene models were generated by EVidenceModeler [40]. Functional annotation employed InterProScan (v5.30-69.0) [41] to extract protein domains and Gene Ontology terms for 16 gene sets, with all data accessible via the BRAD database. Homoeologous gene sets and orphan genes across 18 genomes were identified by OrthoFinder (v2.3.11) [42], while GO enrichment analysis was conducted using TBtools (v1.055) [43].

### Detection of sequence and structural variants between YAXY and T84-66 genomes

Whole-genome alignment between ‘YAXY’ (query) and ‘T84-66’ (reference) was conducted using SyRI [44]. Assemblies were aligned with MUMmer4 nucmer (--mum -t 16) and filtered by delta-filter (identity ≥90%, length ≥ 15 kb). Coordinates were extracted with show-coords. Structural variants (inversions, translocations, duplications) and sequence differences (SNPs, insertions, deletions, copy-number variants, highly diverged regions, tandem repeats) were annotated by SyRI with T84-66 as reference (-r) and YAXY as query (-q). Summary statistics are reported in Table S8.

### Reads alignment and variation detection

Whole-genome resequencing was performed on 213 *B. juncea* accessions (203 *tumida* and 10 other *B. juncea* types), generating 10.79 Tb of raw data. To eliminate potential technical artifacts, stringent quality control was applied to filter low-quality data and adapter sequences by removing: (i) reads containing >10% undetermined bases (N); (ii) reads with >50% bases having a Phred quality score (Q) ≤ 5; (iii) reads contaminated with adapter sequences; and (iv) PCR duplicates generated during library construction. After filtering, 10791.11 Gb of high-quality clean data were retained, with data quality assessed using FastQC (v0.12.1) [45].

High-quality paired-end reads were aligned to the ‘YAXY’ reference genome using the BWA-MEM algorithm (v0.7.17) [46], producing alignment files in BAM format. SAMtools (v1.15) was then used for format conversion, sorting, indexing, and removal of potential PCR duplicates [47]. Joint variant calling of SNPs and InDels across the population was conducted using GATK’s HaplotypeCaller and VariantFiltration modules (v4.2.6.1) under default parameters [48]. Reliable variants were identified by applying GATK’s recommended hard filtering strategy.

Further variant filtering was performed using PLINK (v1.90) with parameters: minor allele frequency (MAF) > 0.05, retention of biallelic sites only (--biallelic-only), and genotype missing rate < 0.20 (-geno 0.20) [49]. Genotype imputation for missing data was executed using Beagle (v5.4) with default settings [50]. Finally, functional annotation of filtered variants—including variant type, genomic region, and predicted functional impact—was performed using SnpEff (v5.1) [51].

### Population genetic analysis

To elucidate the genetic structure and phylogenetic relationships of the *tumida* population, this study employed a multidimensional analytical approach. Based on the filtered high-quality SNP dataset, a Neighbor-Joining tree was first constructed using MEGA7 software (v7.0) [52], followed by visualization via the iTOL online platform (https://itol.embl.de/). Eigenvectors and eigenvalues were calculated using Plink (v1.90), and PCA was visualized using the R programming language. Significant principal components were subsequently incorporated as covariates in downstream association analyses.

The genetic relatedness matrix between individuals was computed using the Normalized_IBS model in TASSEL software (v5.2.40). Heatmaps illustrating the distribution of genetic distances within the population were generated using the R package pheatmap (v1.0.12) [53]. Population genetic structure was analyzed using ADMIXTURE software (v1.30) with parameters set as a cross-validation error threshold (-C 0.01) and parallel computing threads (-j24). The ancestral component proportions derived from ADMIXTURE were visualized using TBtools (v1.123) [43, 54]. Copy number variations (CNVs) in the *tumida* population were detected using the MSeq-CNV algorithm. This approach performs multi-sample joint analysis by simultaneously integrating coverage depth (modeled with a Poisson distribution) and discordant read pair ratios (modeled with a Beta distribution), with critical parameters set at a 200-bp sliding window, posterior probability threshold >0.8, and minimum mapping quality (Q) ≥30 [55].

Genetic variation levels within and between subpopulations were quantified by calculating the nucleotide diversity index (π) and genetic differentiation index (*F*st) using VCFtools (v0.1.16) [56]. For phylogenetic tree construction, 10 *B. rapa* accessions with close ancestral relationships to mustard were selected as the outgroup. A phylogenetic tree was generated using the p-distance method in MEGA7 to clarify the evolutionary relationships among the studied materials.

### Identification of selective sweep signals

To identify genomic regions under selection during the evolution of *tumida*, a selective sweep analysis was performed using fixation index (*F*st) and nucleotide diversity (π). *F*st and the ratio of nucleotide diversity (leaf mustard/stem mustard) were calculated using VCFtools (v0.1.16) with a sliding window approach (20 kb window size, 10 kb step size). The distributions of *F*st and π ratio were log-transformed, and genomic windows ranked in the top 5% of both log-transformed metrics were defined as putative selective regions.

### Genome-wide association analysis of agronomic traits

Based on whole-genome variant data from 203 accessions of *tumida* (comprising 1 382 166 high-quality SNPs and 270 132 InDel markers, with MAF > 0.05 and missing rate < 0.2), GWAS were performed for 21 target traits. All GWAS result files have been deposited in the Figshare repository. The Linear Mixed Model (LMM) was implemented using GEMMA software (Genome-wide Efficient Mixed Model Association, v0.98.3) to correct for genetic background confounding effects [57]. This was achieved by incorporating a kinship matrix (K matrix) as a random effect, combined with the top five principal components (PCs) of population structure as fixed-effect covariates in the model. For model parameterization, the genetic relatedness matrix was calculated using the standardized method. The covariate file included an intercept term by default and integrated PCs to control for population stratification effects.

The significance threshold was optimized via Bonferroni correction by calculating the effective number of independent SNPs (Me) using GEC software (v0.2) [58], with significance defined as *P* = 4 × 10^−8^. Given the relatively weak genetic effects observed for most traits in *tumida*, a dual-threshold criterion was adopted: the primary significance threshold was set at *P* < 1 × 10^−6^ (based on Me correction), and a secondary threshold of *P* < 1 × 10^−4^ was used to identify potential association signals. Manhattan plots and quantile-quantile (QQ) plots were generated using the R packages CMplot (v4.4.1) and qqman (v0.1.9) within the R environment (v4.2.0), visualizing the genome-wide distribution of SNP association significance and the goodness-of-fit of the model, respectively.

### RNA extraction and sequencing library construction

The plant materials for transcriptome sequencing (SPT-H and SPT-L) were cultivated in October 2023 at the Southeast Chongqing Academy of Agricultural Sciences. Samples were collected at 10 days after the onset of stem swelling 10 DAS, 20 DAS, and 30 DAS, snap-frozen in liquid nitrogen, and stored at −80°C until RNA extraction and sequencing.

Total RNA was extracted from plant stalk tissues using the RNAprep Pure Plant Kit (Tiangen), with concentration and purity measured by NanoDrop 2000 (Thermo Fisher Scientific) and integrity assessed by Agilent Bioanalyzer 2100 system (Agilent Technologies). Sequencing libraries were prepared using the Illumina NovaSeq platform to generate 150 bp paired-end reads. The workflow included: (i) mRNA enrichment with oligo(dT) magnetic beads; (ii) double-stranded cDNA synthesis, end repair, and adapter ligation using Hieff NGS Ultima Dual-mode mRNA Library Prep Kit (Yeasen Biotechnology); (iii) library purification with AMPure XP beads (Beckman Coulter) followed by quality control on Agilent 2100 system.

### Transcriptomic data analysis

Transcriptome sequencing and analysis was performed for two distinct purposes: (i) To facilitate genome annotation, RNA sequencing data were generated from the *tumida* accession ‘YAXY’. (ii) To conduct differential gene expression analysis, RNA was extracted specifically from stem peripheral tissues of the contrasting SPT-H and SPT-L accessions and sequenced. Raw reads were processed with fastp (parameters: -z 4 -q 20 -u 30 -n 5) [59]. Cleaned reads were aligned to the corresponding reference genome using HISAT2 (v2.2.0) [60]. Gene expression levels were quantified as TPM (transcripts per million) via StringTie (v2.1.3b) [61].

### Allele frequency estimation of *BjuA05g15010* missense variants

To ensure cross-locus comparability, per-group allele frequencies were estimated on the common effective sample set, defined as accessions with non-missing genotypes at both SNPs (ChrA05: 9741792 and 9 743 180). Within this set, allele counts (AC), total alleles (AN), and allele frequencies (AF = AC/AN) were computed under the diploid assumption, yielding ALT_AF and REF_AF (REF_AF = 1 − ALT_AF). REF/ALT were defined relative to the YAXY reference genome. The full summary is provided in Table S20.

## Supplementary Material

Web_Material_uhaf298

## Data Availability

The newly generated raw sequence data reported in this paper have been deposited in the Genome Sequence Archive (Genomics, Proteomics & Bioinformatics 2021) in National Genomics Data Center (Nucleic Acids Res 2022), China National Center for Bioinformation/Beijing Institute of Genomics, Chinese Academy of Sciences (GSA: CRA025655, CRA026508, CRA026501 and CRA025656) that are publicly accessible at https://ngdc.cncb.ac.cn/gsa. Publicly available resequencing data supporting this study include NCBI SRA PRJNA615316 and PRJNA1148674. For expression analysis of *BjuA05g15010* between *tumida* and *integrifolia*, we used data from 22 *integrifolia* samples (PRJNA544908, PRJNA672814, PRJNA800112) and 19 *tumida* samples (PRJNA289188, PRJNA477240, PRJNA878553). Genome assembly files, annotated protein-coding genes, and GWAS-related file are available in the Figshare repository (https://figshare.com/s/7e8cc1eb750c4c20add7).
