# Supplementary material for: Population genomic insights into the domestication of Brassica juncea var. tumida
Source: Hortic Res. 2025 Nov 5;13(2):uhaf298. doi: 10.1093/hr/uhaf298 (PMC12933663; doi:10.1093/hr/uhaf298)
Supplement: Web_Material_uhaf298 [file web_material_uhaf298.zip › Supp_Fig.docx]

**Supplementary Figure**


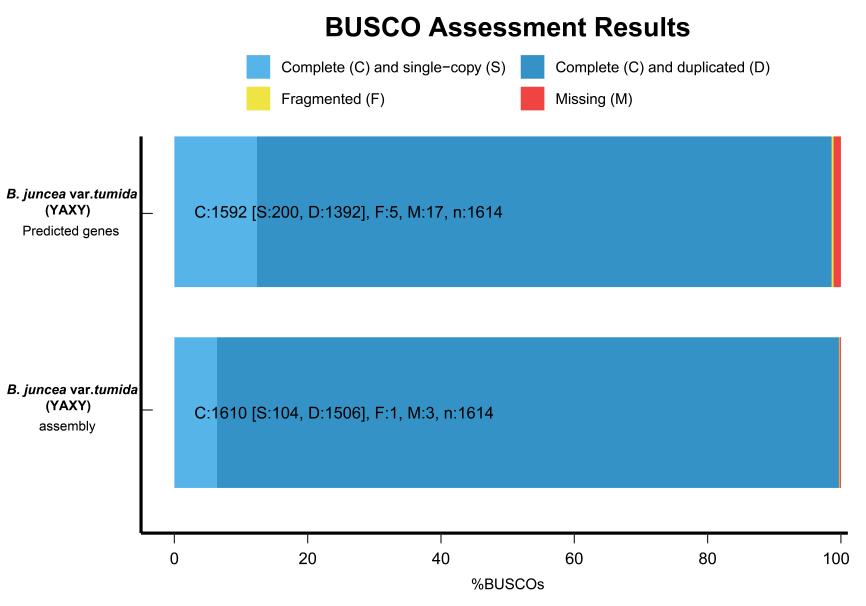


**Figure S1.** BUSCO assessment of genome assembly and gene prediction quality for *B.* *juncea* var. *tumida* (YAXY) .

BUSCO (Benchmarking Universal Single-Copy Orthologs) assesses genome completeness by classifying genes as Complete (C), Single-copy (S), Duplicated (D), Fragmented (F), or Missing (M). For *B. juncea* var. *tumida* (YAXY), the genome assembly contained 1610 complete BUSCOs (104 single-copy, 1506 duplicated), 1 fragmented, and 3 missing out of 1614 total. The predicted gene set contained 1592 complete (200 single-copy, 1392 duplicated), 5 fragmented, and 17 missing, indicating high completeness in both assembly and annotation.


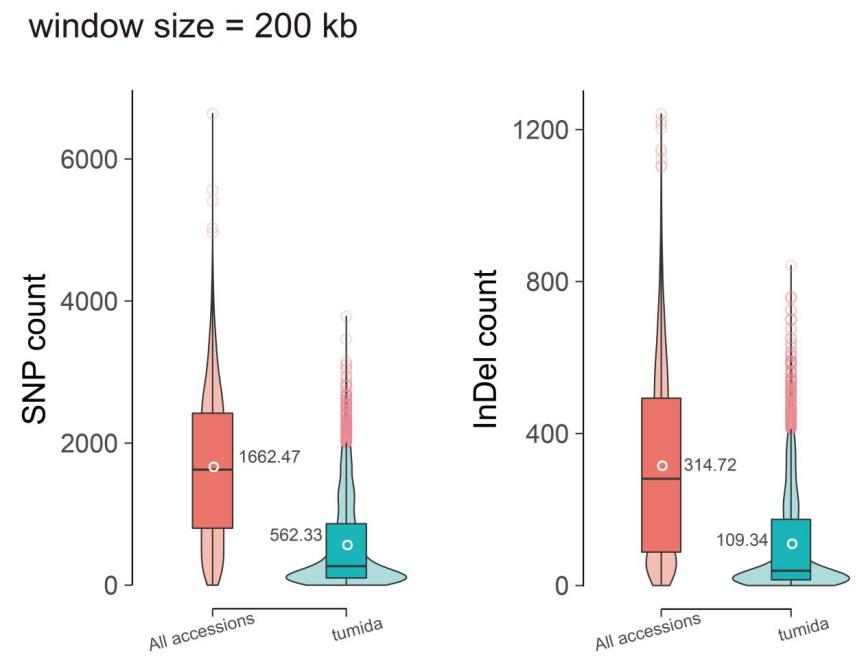


**Figure S2.** Comparison of SNP and InDel distributions between *tumida* and all *B. juncea* accessions (window size = 200 kb).

The left panel shows single nucleotide polymorphism (SNP) counts and the right panel shows insertion–deletion (InDel) counts. Compared with all accessions, the *tumida* group exhibited markedly lower mean SNP counts (562.33 vs. 1662.47) and mean InDel counts (109.34 vs. 314.72), indicating reduced genetic variation consistent with a bottleneck in *tumida*.


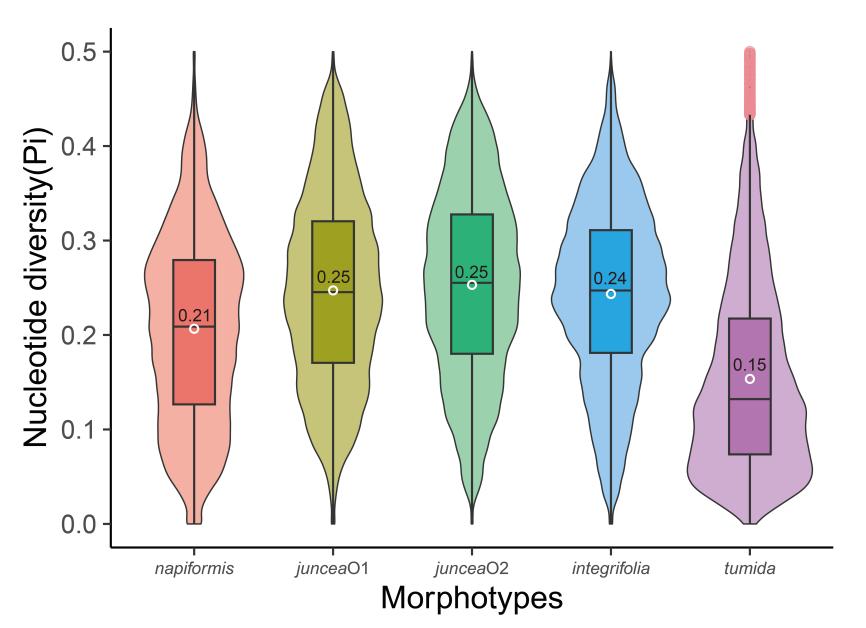


**Figure S3.** Nucleotide diversity of *B. juncea* subgroups using the Purple-leaf Mustard (PM) reference genome.

Violin and box plots show the distribution of nucleotide diversity (π) in five *B. juncea* morphotypes: *napiformis*, *juncea* O1, *juncea* O2, *integrifolia*, and *tumida*. Using *B. juncea* var. Purple-leaf Mustard as the reference genome, we mapped 717 accessions and calculated nucleotide diversity with the same methods applied in the main text. The numbers within each plot indicate mean π values. *Tumida* exhibited the lowest nucleotide diversity (0.15), significantly lower than the other morphotypes (0.21–0.25), suggesting that *tumida* experienced a genetic bottleneck during its evolutionary history.


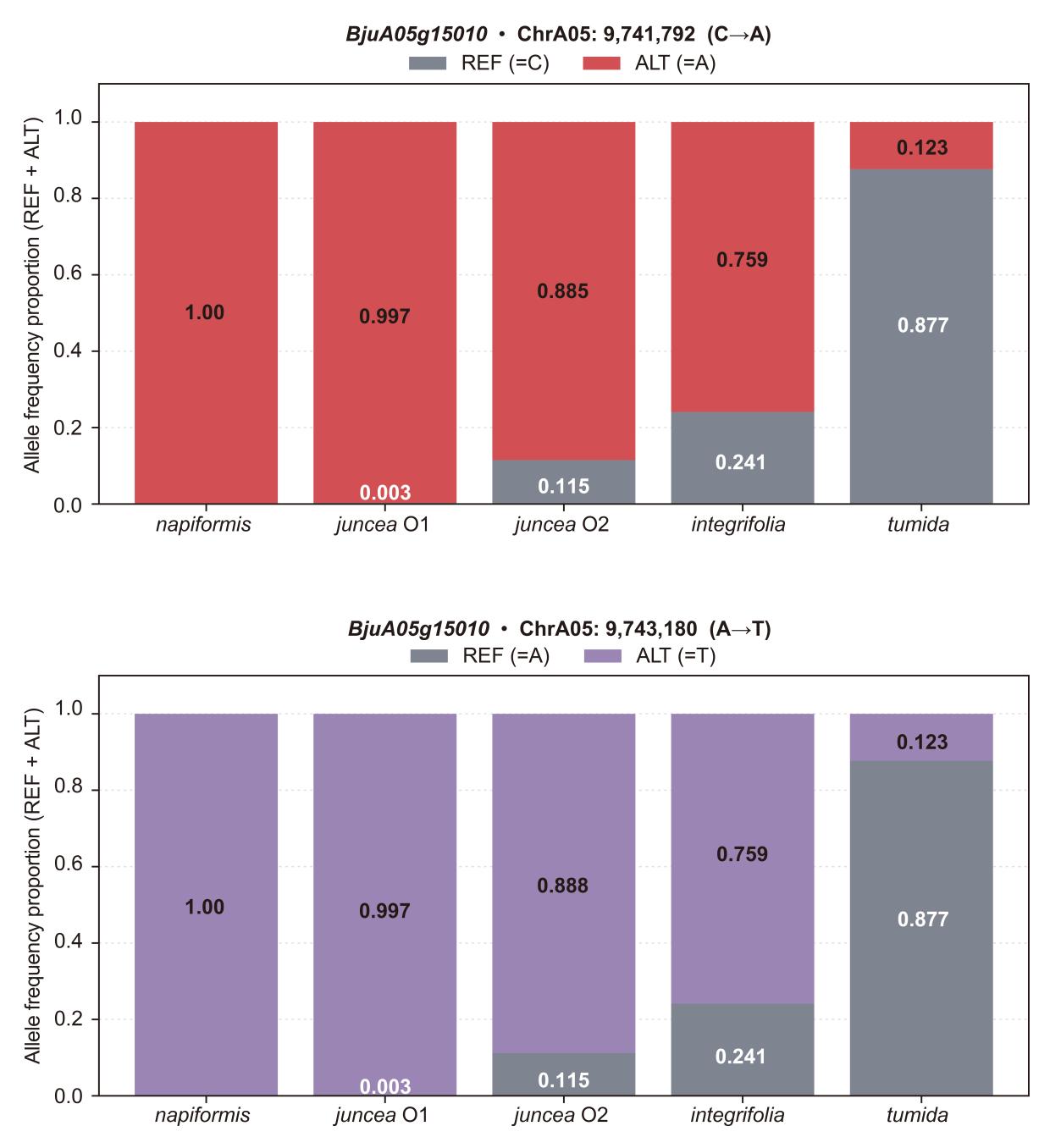


**Figure S4.** Allele frequency distribution of two key SNPs in the *BjuA05g15010* gene across five *B. juncea* groups. Bar plots show allele frequencies at ChrA05:9,741,792 (C→A) and ChrA05:9,743,180 (A→T) in *napiformis*, *juncea* O1, *juncea* O2, *integrifolia*, and *tumida*. The y-axis represents allele frequency proportion (REF + ALT = 1). To ensure comparability across groups, only the common set of individuals with valid genotypes at both SNPs was used (effective N: *napiformis* = 26, *juncea* O1 = 173, *juncea* O2 = 130, *integrifolia* = 112, *tumida* = 219). REF alleles are shown in gray, while ALT alleles are shown in red (A at ChrA05:9,741,792) or purple (T at ChrA05:9,743,180). Numbers inside the colored bars indicate the frequency of REF and ALT alleles for each group.

Population variant analysis identified two missense mutations in *BjuA05g15010*: ChrA05: 9,741,792 (C→A, H6Q) and ChrA05: 9,743,180 (A→T, I414L). Allele frequency analysis revealed that these missense alleles are present at high frequency in ancestral non-swollen lineages—*napiformis* (ALT AF=1.000/1.000), *juncea* O1 (0.997/0.997), *juncea* O2 (0.885/0.888), and *integrifolia* (0.759/0.759)—but occur at low frequency in *tumida* (0.123/0.123).

**
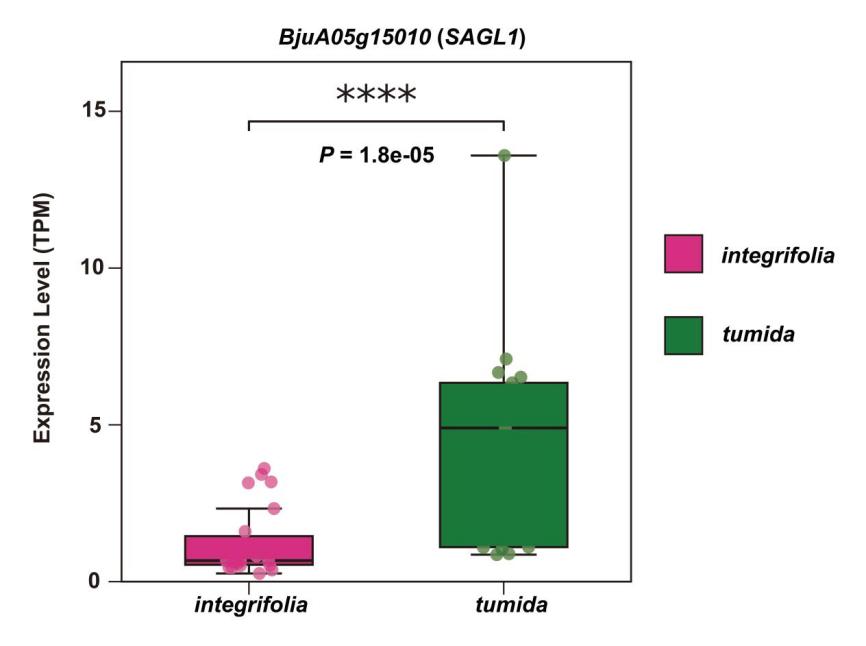
**

**Figure S5.** Expression Levels of *BjuA05g15010* and CK2B1–E2Fa Homologs in YAXY between *integrifolia* and *tumida*. Statistical significance was assessed using the two-sided Mann–Whitney U test. Sample sizes: *integrifolia* (n = 22), *tumida* (n = 19). Significance codes: **** *P* < 1 × 10⁻⁴; *** *P* < 1 × 10⁻³; ** *P* < 1 × 10⁻²; * *P* < 0.05; n.s., not significant (*P* ≥ 0.05). Pink bars represent *integrifolia*, and green bars represent *tumida*.

Based on publicly available RNA-seq datasets (PRJNA544908, PRJNA672814, PRJNA800112, PRJNA289188, PRJNA477240, and PRJNA878553), TPM quantification showed that *BjuA05g15010* was expressed at significantly higher levels in *tumida* than in *integrifolia* (*P* < 1 × 10⁻⁴).
